# Thermal Load of Mine Rescuer in the Underwear and Protective Clothing with Phase Change Materials in Simulated Utility Conditions

**DOI:** 10.3390/ma13194320

**Published:** 2020-09-28

**Authors:** Grażyna Bartkowiak, Anna Marszałek, Anna Dąbrowska

**Affiliations:** 1 Department of Personal Protective Equipment, Central Institute for Labour Protection–National Research Institute, Wierzbowa 48, 90-133 Lodz, Poland; andab@ciop.lodz.pl; 2 Department of Ergonomics, Central Institute for Labour Protection–National Research Institute, Czerniakowska 16, 00-701 Warsaw, Poland; anmar@ciop.pl

**Keywords:** protective clothing, phase change materials, PCM, heat load, mine rescuer

## Abstract

A new set of underwear and protective clothing with phase change materials (PCM) for mine rescuers has been developed in order to increase their safety of work. It includes PCM pouches absorbing excess heat from the body. In order to evaluate thermal load of mine rescuers, physiological tests were carried out for three variants of possible use of the developed set of clothing: for mine rescuers wearing only the underwear with PCM; for a set of underwear and protective clothing; and for a complete set of clothing with closed-circuit compressed oxygen breathing apparatus. Tests were performed in difficult microclimate conditions, reflecting the typical working conditions of rescuers that cause a significant thermal load. The use of outer clothing shortened safe time of exposure to such conditions by about 36%, while the addition of respiratory protective device to this set further shortened this time to a lesser extent (by another 13%).

## 1. Introduction

Underground mining is characterized by difficult geological and mining conditions. They are accompanied by many natural hazards such as: rock bursts, gas and rock outbursts, methane atmosphere, fires and coal dust explosions, and flooding with water [1]. Coal mining is gradually taking place at ever greater depths—each year it increases by an average of about 8 m, which results in an intensification of the climatic threat [2]. In the near future, mining works will be carried out in a rock mass with a temperature reaching 50 °C, and the climatic threat may prove to be one of the basic threats determining the safety of miners and the possibility of carrying out works.

In the coal mining industry, there is still a high probability of accident events. They cover the operation of large spaces and are characterized by the strong intensity of the course. They damage excavations and technical infrastructure, often leading to catastrophic events and causing loss of life or serious injuries among miners. Considering the high accident rate and threats occurring in the coal mining industry, attention should be paid to the difficult and very dangerous conditions for conducting rescue operations, which take place in extreme climatic conditions (at dry temperature reaching even T_s_ = 35–45 °C, relative humidity = 70–97%, air velocity v = 0.5–2.5 m/s) with the possibility of fire and explosion hazards [3,4,5]. Besides extremely hard environmental conditions, mine rescuers are subjected to high physical effort during rescue work, which is additionally contributed by the total weight of the equipment [6,7]. Basic mine rescuers’ equipment consisting of complete clothing (about 6 kg), respiratory equipment (15 kg), lamps, and basic devices causes an additional load of about 25 kg. In the case of transporting injured persons on the stretcher, the average additional load can be even about 45–50 kg/person. Dry complete clothing is already 25% of the primary load of the rescuer, this load increases during operation, as moisture (or dipping, while overflowing water droplets) is an additional ballast increasing the energy expenditure.

The clothing used so far by mine rescuers have not met their needs in terms of comfort of use. In order to remove excess heat from the body, an additional element of clothing was used in the form of cooling vests, which, however, constituted an additional load, had a limited cooling time not adjusted to the length of rescue operations, and did not meet the requirements for protection against thermal factors, flame, and static electricity. Taking into account difficult environmental conditions in which rescue operations are performed, the appropriate selection of fabrics for protective clothing and its construction in order to prevent excessive heat accumulation in a mine rescuer’s body are of the highest importance [8]. The weight of clothing should be reduced and a material should be applied that can easily transport moisture to the environment and does not absorb large amounts of water when wet.

The above factors constituted the premise for the development of a set of underwear and protective clothing for mine rescuers, designed taking into account their requirements and needs, as well as conditions of conducting rescue operations. Therefore, within a research project carried out by a consortium consisting of a research institute specialized in the field of safety at work, end-users (mine rescuers), and a manufacturer of protective clothing, the set of undergarment and protective clothing was developed. It was assumed that underwear and protective clothing for mine rescuers should ensure safety and allow heat release under conditions of rescue work in a hot environment to prevent overheating of the body.

The aim of this research is to introduce a new set of underwear and protective clothing for mine rescuers, as well as evaluate the influence of selected protective equipment on the mine rescuers’ thermal load in climatic conditions similar to those that occur during rescue operations.

## 2. Protective Underwear and Clothing with Phase Change Materials for Mine Rescuers

Due to the hazardous environment in which mine rescuers perform their operations, their clothing should protect against thermal factors and static electricity, as well as support human thermoregulation to prevent from overheating. Therefore, such clothing should meet essential requirements of Regulation (EU) 2016/425 [9] on the personal protective equipment and relevant standards, i.e., EN ISO 11612:2015 [10], which relates to a flame-retardant protective clothing, and EN 1149-5:2018 [11] for the anti-static protective clothing intended to use in an explosive atmosphere.

Based on the analysis of the course of rescue operations with an indication of explosion and climate hazard zones, it was found that significant sections of mines are without threats and there are also sections where these threats occur individually. For this reason, the protective clothing and underwear for mine rescuers were designed to enable its quick configuration (in order to easily adapt it to the type of dominant threat). In the section of without threats or only with a climatic threat, a minimum amount of clothing or clothing with open zippers is expected. On the other hand, the complete protective clothing should be used in the section with direct risk of flame and explosion. If there are associated hazards (climatic hazard and low risk of explosion) in a given mine’s section at the same time, the clothing should be configured to protect the mine rescuer’s body with at least one non-flammable protective layer, and maximally unzip all ventilation openings to ensure the possibility of heat dissipation by evaporation of sweat. It is important that the clothing also protect the head and neck.

Given the severe climate conditions during the rescue operations, as well as the considerable physical effort, a set of underwear and clothing must support the body’s thermoregulation and be as light as possible to enable the evaporation of sweat and heat transfer.

### 2.1. Phase Change Materials

The underwear and protective clothing with phase change materials (PCM) were designed in order to prevent from mine rescuers’ overheating. PCM were used to collect excess heat generated by the rescuer, which uses the endothermic process of transformation from the solid to liquid form. The introduction of PCM into the clothing structure, characterized by a phase transition temperature at a level similar to the temperature of the skin in a comfort state, causes that, as a result of heat accumulation in the body and an increase in the temperature of user skin, the PCM begin to melt, while receiving heat released by the clothing user’s body. When choosing PCM to be used in the protective clothing and underwear, it was assumed that they should have a melting point resulting from the temperature distribution in clothing structure in predicted utility conditions. The PCM melting temperature was chosen, so that only the temperature increase in the undergarment microclimate would activate the melting process of subsequent phase change materials, thus extending the effective cooling time and the amount of heat received from the undergarment microclimate. Based on the analysis of conditions of rescue operation, PCM with a melting temperature between 32–37 °C were selected for the use in the protective clothing and underwear, which will ensure cooling in ambient temperature of about 30 °C to about 40 °C. An important parameter characterizing the phase transition materials is enthalpy, which allows to determine how much heat is absorbed or generated by PCM during the transition from the solid to a liquid form or vice versa. The higher the enthalpy value, the more heat can be removed from the undergarment microclimate, and it is recommended that this value is not less than 100 J/g. An important aspect of designing underwear with cooling elements is also the choice of phase transformation material form and the mass introduced into the underwear structure. Most of the PCM available on the market are paraffinic compounds that are flammable.

Studies have shown that PCM used in clothing products in the form of microcapsules have a very low ability to receive excess heat and are insufficient to ensure the reduction of thermal discomfort in a hot work environment [12,13,14,15]. Textile products, in which PCM macrocapsules are placed, have a greater ability to receive excess heat [2]. When designing cooling systems using phase change materials, PCM parameters should be selected to suit to the environmental conditions of predicted use. Studies analysing the effectiveness of designed PCM products in reducing heat load in hot and even extremely hot (corresponding to fire) microclimate have shown that the selection of proper enthalpy and phase change temperature is very important [15,16,17].

Due to the above requirement of non-flammable PCM, the temperature of phase change and enthalpy macrocapsules were used in the underwear and clothing for mine rescuers, in which the PCM are enclosed in a non-flammable, thin polymer shell. Characteristics of those PCM are presented in Table 1.

**Figure 1:**
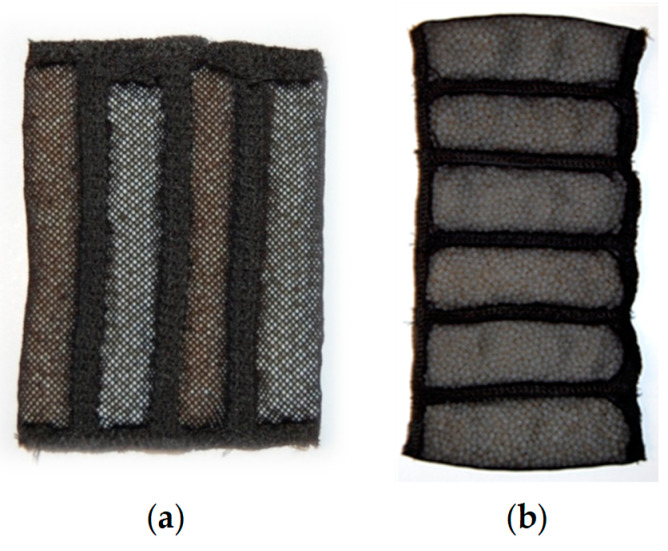
View of the cooling elements with PCM microcapsules (**a**) used in underwear—weight about 30 g, (**b**) used in clothing—weight about 169 g in the case of six-channel pouch and 44 g in the case of four-channel pouch.

Cooling elements containing PCM are prepared in the form of rectangular pouches with four or six channels, in which macrocapsules of MPCM 32 and MPCM 37 (Microtek Laboratories Inc, Moraine, OA, USA) are alternately introduced (Figure 1). The form of pouches was designed separately for the underwear and protective clothing application in order to adjust them to the rest of equipment and avoid generation of discomfort due to the pressure, as well as to support absorbing the excess heat from the body as much as possible.

### 2.2. Protective Underwear with PCM

**Figure 2:**
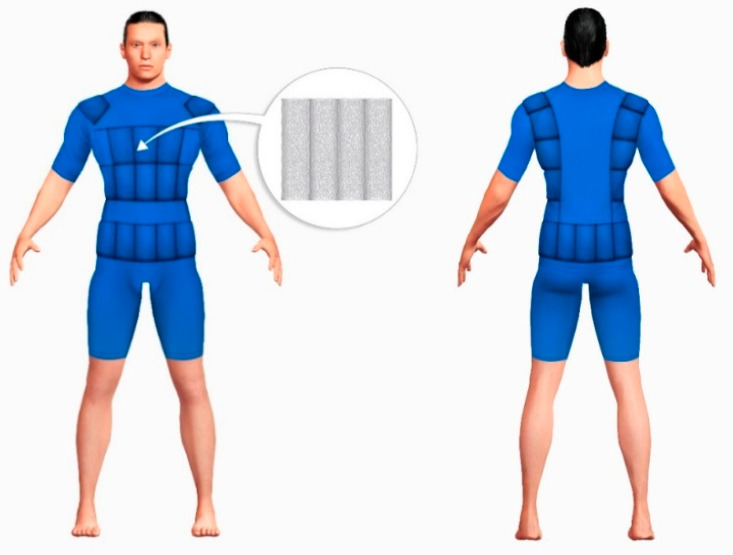
A scheme of protective underwear with PCM elements.

The protective underwear (Figure 2) has been made from a knitted fabric (90% Lenzing FR, 8% p-aramid, 2% antistatic fibre) with mass per square meter equal to 193.5 g/m^2^. The selection of fabric was made on the basis of results of laboratory testing of the protective properties, as well as thermal comfort related properties. The selected fabric meets the requirements of harmonized standard EN ISO 11612: 2015 [10] and EN 1149−5: 2018 [11]. Test results indicate that the fabric meets the requirements for non-flammability (code A1 and A2 according to EN ISO 11612: 2015 [10]), both when new and after 50 washing cycles. The fabric meets also the criteria for antistatic properties, charge decay time t_50_ < 0.01, and the shielding factor S = 0.666 (according to requirements of EN 1149−5: 2018 [11]) after 50 washing cycles. Based on the laboratory tests, it has been found that the selected knitted fabric is characterized by the low water vapour resistance (2.89 m^2^Pa/W), which has a positive influence on shaping undergarment microclimate in terms of drawing sweat from the human body.

The underwear was in the form of a T-shirt and shorts. Cooling elements made of PCM were placed in the underwear, so that they cover areas of the body that are subject to the greatest heating, i.e., on the torso and on the back. They are introduced to the underwear by means of specially designed pockets that make the PCM elements detachable. The pockets are also sewn in a way preventing the PCM elements from falling out.

The arrangement of cooling elements in the underwear allows heat to be removed from the parts of the body that are the most heated during the exercise. The arrangement of PCM elements on the torso affects the efficiency of their operation [17], but in the case of underwear for rescuers, the equipment that is worn on clothing was of fundamental importance. To ensure the compatibility of underwear design with other equipment of mine rescuers, and in particular with the respiratory protective equipment, cooling elements were placed in pressure-free areas (in total 860 g of phase change materials were introduced).

PCM elements have been tested for the inflammation resistance and they meet the requirements of harmonized standard EN ISO 11612: 2015 [10].

### 2.3. Protective Clothing

In order to provide higher protection level when an increased risk of fire of explosion exists, mine rescuers should be additionally equipped with outer protective clothing. The developed protective clothing for mine rescuers consists of a blouse, bib, and brace trousers. The clothing has been made of woven fabric (93% meta-aramid fibers, 5% para-aramid fibers, and 2% antistatic fibers). The selection of fabric was made on the basis of the laboratory results of testing the protective properties as well as thermal comfort related properties. The selected fabric meets the requirements of harmonized standard EN ISO 11612: 2015 [10] and EN 1149−5: 2018 [11]. Test results indicate that the fabric meets the requirements for non-flammability (code A1), both when new and after 50 washing cycles. The fabric meets also the criteria for antistatic properties: charge decay time t_50_ < 0.01, and the shielding factor S = 0.813, after 50 washing cycles. It is characterized by a relatively low value of the surface mass (155 g/m^2^), which is important for comfort and yet satisfactory strength (breaking force 950 N in the longitudinal direction and 880 N in the transverse direction). Also, the tear strength for the selected fabric is quite high, around 50 N before washing and around 35 N for both directions after maintenance. The fabric is also abrasion resistant (over 100,000 abrasion cycles). Water vapour resistance after 50 maintenance cycles was 4 m^2^Pa/W, which is quiet good value in relation to comfort.

**Figure 3:**
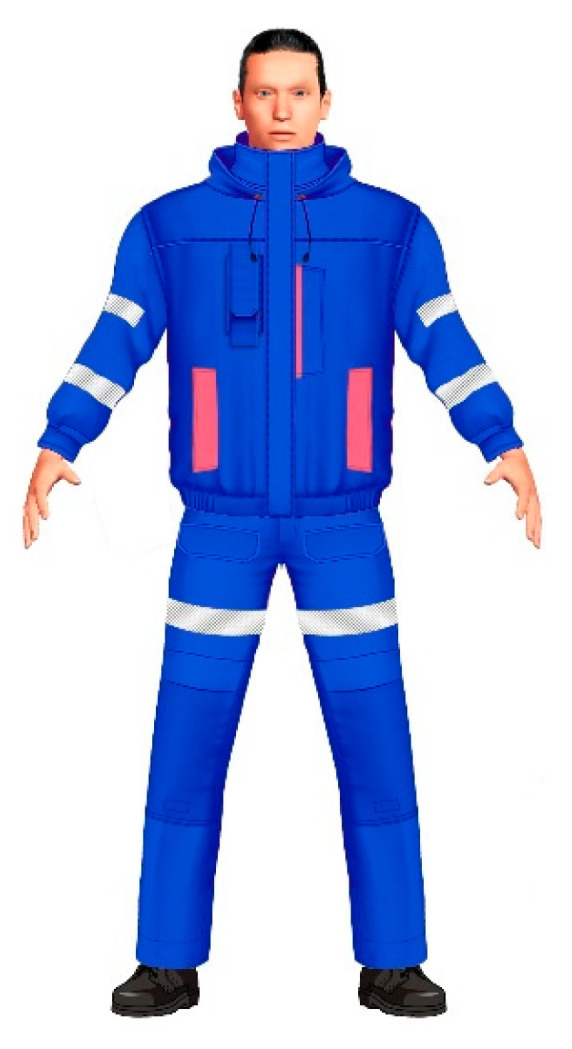
A scheme of the protective clothing for mine rescuers.

The scheme of the developed protective clothing for mine rescuers is presented in Figure 3. The design of the developed clothing ensures compatibility with other equipment worn by mine rescuers. Arrangement of pockets is adjusted to rescuers’ needs in order to ensure convenient access to them even during use of a breathing apparatus.

**Figure 4:**
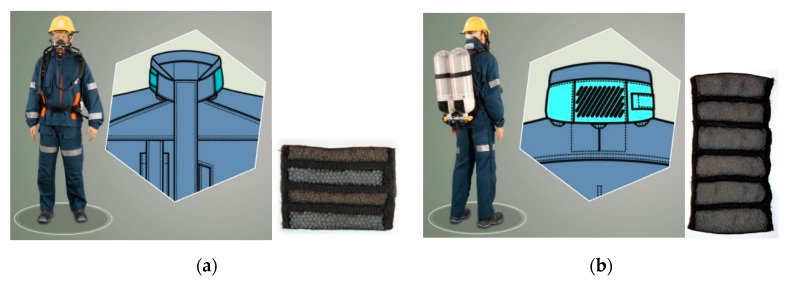
Location of cooling elements in the stand-up collar: (**a**) four-channel cooling element (to be placed in the side pockets of the stand-up collar), (**b**) six-channel cooling element (to be placed in the back stand-up collar).

Moreover, PCM pouches are introduced in the stand-up collar (Figure 4) and on the back of the jacket in order to provide additional support of thermoregulation processes. On the lateral side of the sleeve, along the torso, and on the lateral side of the leg, vents have been also introduced in order to increase drawing the excess heat from the rescuer’s body. The total weight of PCM introduced to the protective clothing is about 257 g.

## 3. Research Methodology

### 3.1. Characteristics of Volunteers and Test Variants

The tests were performed with the participation of six mine rescuers. They were 34.5 ± 6.2 years old, with a body weight of 82.0 ± 4.8 kg, and maximal aerobic capacity of 48.2 ± 5.2 mL O_2_/kg/min. Test participants were selected on a basis of qualification examinations including current state of health and physical examination. Moreover, cardiac stress test, 24-h Holter ECG monitoring, spirometry, and physical work capacity (PWC_170_) test were additionally performed.

**Figure 5:**
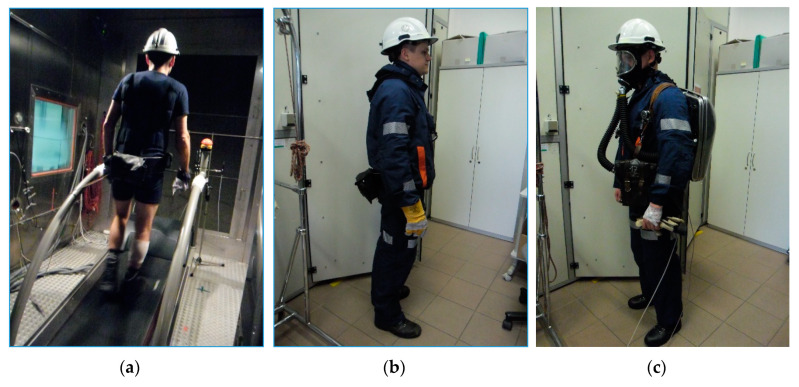
A view of the mine rescuer wearing the tested clothing variant: (**a**) BA, (**b**) BA OW, (**c**) BA W70.

In order to provide climate conditions similar to those in real rescue operations, tests were carried out in a climatic chamber in air temperature of 32 °C, air velocity of 1.0 m/s, and relative humidity of 80–85%. The paper presents results obtained for three test variants differed by the personal protective equipment used. The test variants are listed in Table 2, and a view of the mine rescuer wearing the tested clothing variant is shown in Figure 5. The variants used in the research reflect the configuration possibilities of the developed set of clothing and underwear during work performed by rescuers depending on the level of hazards.

During the tests, mine rescuers were equipped with a helmet, a lamp, and gloves. Moreover, in test variant BA W70, they additionally used breathing apparatus W-70 (“FASER” S.A., Tarnowskie Góry, Poland), which is the basic equipment of Polish mine rescue services, used as a respiratory protective device (RPD). W-70 operates in a closed circuit, what means that it ensures a complete insulation of the user from the surrounding atmosphere. The selected RPD is a negative pressure device that operates in a closed-circuit breathing system, with oxygen compressed in a steel cylinder. The protection time provided by this RPD is about 4 h depending on the utility conditions. Its weight without the breathing mask is about 14 kg [18]. During the tests, a SAT-2M inhaled air cooler was added so the weight of W-70 at the beginning of the tests was 22.94 ± 0.10 kg.

### 3.2. Testing Procedure

The assumed time for the complete test was 120 min; however, it was limited when either thresholds for physiological parameters such as internal temperature (measured in the gastrointestinal tract T_abd_) and heart rate (HR) were achieved or subjective signs of fatigue were notified. In order to guarantee safety of test participants, the following limits were adopted [19]: 38.5 °C and > 85% HR_max_, respectively_._ Before the tests, the volunteers signed the approval for participation in the study. Moreover, a physician was present during the tests.

Each experiment was preceded with drinking 500 mL of water and swallowing the sensor for internal temperature monitoring. Then, other relevant sensors were mounted, i.e., sensors for monitoring skin temperature, air temperature, and relative humidity in the underclothing microclimate, ECG electrodes, and heart rate recorder (Polar Electro Oy, Kempele, Finland).

In the next step, the participant put on the clothing that included the underwear (without PCM pouches), protective clothing, socks, and boots. In such clothing, they stayed for 45 min in air temperature 21 °C and relative humidity 50% in order to ensure equal initial conditions for all tests.

When the participants were thermally stabilized, they changed the underwear into the one filled with PCM pouches. In the case of the test variant with the protective clothing, the jacket was also changed to that with the PCM pouches introduced to the collar in order to provide full assumed thermoregulation effect of PCM during the tests (i.e., not limited by preparation activities). Finally, other necessary equipment was provided as described in Section 3.1.

After preparation, participants moved to the climatic chamber with air temperature 32 °C, where they performed assumed physical activity on a treadmill. It corresponded to 25% of physical endurance and was individually determined for each participant. The participants walked on the treadmill with a constant speed of 3 km/h. The physical effort was adjusted by means of inclination angle of the treadmill on a basis of the weight of a fully equipped mine rescuer depending on the test variant to be carried out.

The test procedure was approved by the Ethics Committee of the Medical University of Warsaw, which has granted consent for tests on protective clothing carried out in a climatic chamber with the participation of rescuers under difficult microclimatic conditions.

### 3.3. Measured Parameters

The analysis of physiological reactions of rescuers during the tests was performed on a basis of the following parameters:

Internal temperature measured in the gastrointestinal tract (T_abd_) by means of the Thermometric Physiological Monitoring System (VitalSense, Philips Respironics, Bend, OR, USA);

Local skin temperature and relative humidity above the skin at four locations as per EN ISO 9886:2004 [20] by means of i-Button (Maxim Integrated, San Jose, CA, USA), the average values obtained from those locations for six participants were calculated and presented in figures;

The body and clothing weight prior to and after the test, taking into account the time of experiment, in order to determine the sweating intensity, by means of a platform scale F 150 S-D2 (Sartorius, Gottingen, Germany).

Moreover, during the whole test, the heart rate was monitored by means of both an FX 2000 cardiac monitor (Emtel, Zabrze, Poland) and wireless monitor (Polar Electro Oy, Kempele, Finland). The arterial blood pressure was measured prior to and after the test using medical equipment (Omron, Cau Giay, Vietnam). The average weighted skin temperature (t_sk_) was calculated according to EN ISO 9886:2004 [20] and heat accumulation (S) was determined according to the equation:

S = (3.55 × m_cp_/A_Du_) × (0.9 × Δt_abd_ + 0.1 × Δt_sk_) × T_eks_^−1^ (1)

in which m_cp_—initial body weight, A_Du_—body surface, and T_eks_—exposure duration.

The body surface was established separately for each participant on the basis of Du Bois equation [20]:

A_Du_ = 0.202 × W_b_^0.425^ × H_b_^0.725^ (2)

in which W_b_ —body weight and H_b_—body height.

Selection of the parameters measured were conducted for the purpose of ensuring volunteers’ safety during the experiment, evaluation of level of the thermal load, and estimation of safe time of work in the certain conditions. The obtained measurement data were subjected to statistical analysis. In order to verify normality of distribution, Shapiro-Wilk test was carried out and homogeneity of variance was checked by means of Levene’s test. In case of positive results, statistical significance of differences was checked by analysis of variance (ANOVA) and the lowest significance difference (LSD) test was used as the post-hoc test. If the assumptions for ANOVA were not met, non-parametric Kruskal-Wallis ANOVA and Median Tests were performed. The level of significance of the differences of 0.05 was adopted. The statistical analysis was conducted with the use of Statistica 13.1 software (StatSoft Polska sp. z o.o., Cracow, Poland).

## 4. Results

### 4.1. Test Duration and Reasons for Termination of the Experiment

**Figure 6:**
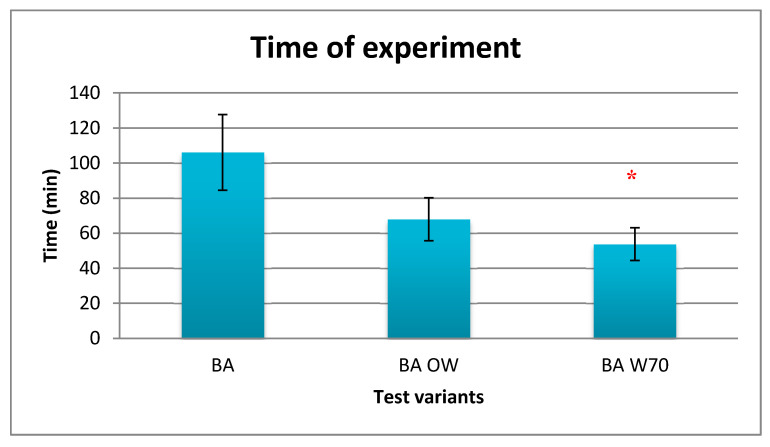
Results of average test duration during the physical exercise at a level of 25% VO_2max_ in air temperature of 32 °C, relative humidity of 80–85%, and air velocity of 1 m/s. * statistically significant difference, *p* < 0.05.

Results of mean test duration are presented in Figure 6, while reasons for termination of the experiments are presented in Table 3.

The average duration of tests varies depending on the tested variant and indicates how the rescuers’ clothing and equipment affect the experiment time. On the basis of the obtained results (Figure 6), it can be stated that the longest mean time of experiment was in the case of the BA test variant (about 106 min), while the shortest one was in the case of the BA W70 test variant (about 54 min). The obtained difference between BA and BA W70 was statistically significant. Due to the limited time of experiment in the case of the BA OW and BA W70 variants, after 60 min of the experiment there was not enough data to conduct statistical analysis.

Analyzing the reasons for the termination of the experiment, it can be noted that only in the case of the BA test was variant time of experiment the reason for termination of the study three times. In the case of experiment with the protective clothing BA OW, reaching limit values of physiological parameters (HR and t_abd_) determined the test duration. Only for the variant BA W70, for one case, did subjective considerations determine the end of experiment.

### 4.2. Heart Rate

**Figure 7:**
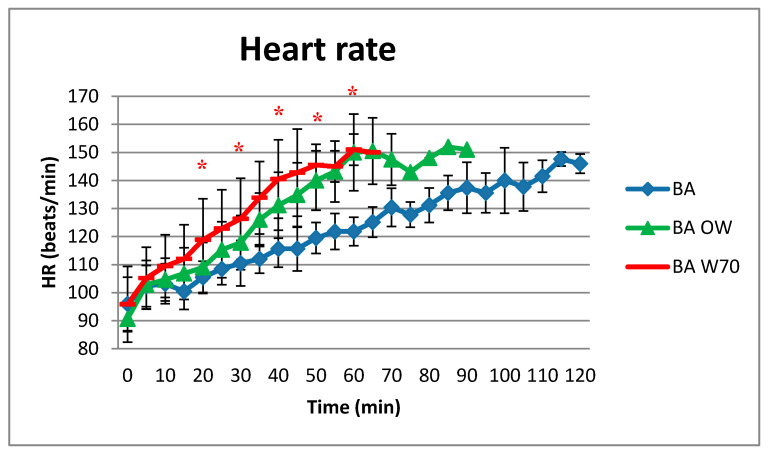
Results of average heart rate during the physical exercise at a level of 25% VO_2max_ in the air temperature of 32 °C, relative humidity of 80–85%, and air velocity of 1 m/s. * statistically significant difference, *p* < 0.05.

Research results regarding changes in the heart rate (HR) are presented in Figure 7.

The changes in HR were the mildest in the variant BA; after the longest-lasting testing, the final HR value was the lowest, reaching the level of 146 beats/min. The HR level was slightly higher for the BA OW (151 bpm) with shorter test time (90 min) and similarly for the BA W70 variant, however in 65 min of the test. Differences in HR for the variants BA and BA W70 were statistically significant between 20 and 60 min, and for the variants BA and BA OW, between 40 and 60 min.

### 4.3. Internal Temperature

**Figure 8:**
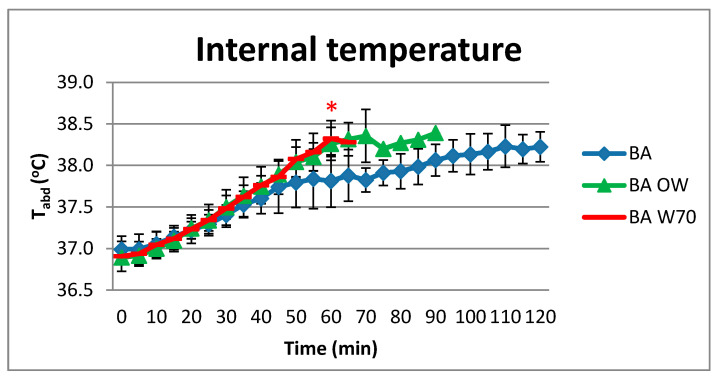
Results of average internal temperature during physical exercise at a level of 25% VO_2max_ in air temperature of 32 °C, relative humidity of 80–85%, and air velocity of 1 m/s. * statistically significant difference, *p* < 0.05.

The test results of internal temperature measurements are presented in Figure 8.

As expected, the lowest internal temperature was observed for variant BA and the results are statistically different from the results for variants BA OW and BA W70. The use of underwear with the protective clothing caused an increase in T_abd_ that was faster than in the conditions only with the underwear (BA). In the 60th minute of the study, it was found that the level of T_abd_ was statistically significantly higher for both BA OW and BA W70 than for the BA variant.

### 4.4. Mean Weighted Skin Temperature

**Figure 9:**
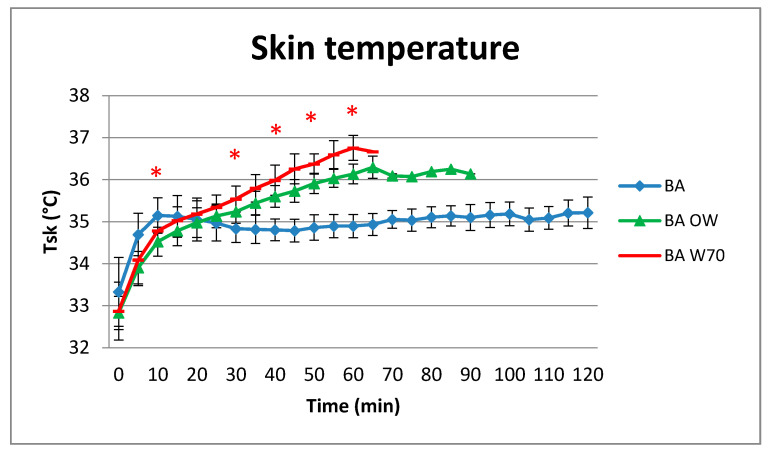
Results of average weighted skin temperature during the physical exercise at a level of 25% VO_2max_ in the air temperature of 32 °C, relative humidity of 80–85%, and air velocity of 1 m/s, * statistically significant difference, *p* < 0.05.

Results concerning the determined mean weighted skin temperature (T_sk_) are presented in Figure 9.

T_sk_ level was statistically significantly lower for the BA variant compared to both BA OW and BA W70 variants from 30 to 60 min of test and also in the 10th min of the experiment between the BA and BA OW variants (Figure 9). For variant BA, the skin temperature was stable at a level of around 35 °C from 8 min to the end of experiment, while for variants BA OW and BA 70 it had been growing steadily since the beginning of experiment. For the BA OW variant, no increase in the skin temperature from 65 min to the end of experiment (90 min) was observed, and skin temperature stayed at a level close to 36 °C. In variant BA W70, the end level of T_sk_ was even greater and close to 36.8 °C.

### 4.5. Relative Humidity above the Skin

**Figure 10:**
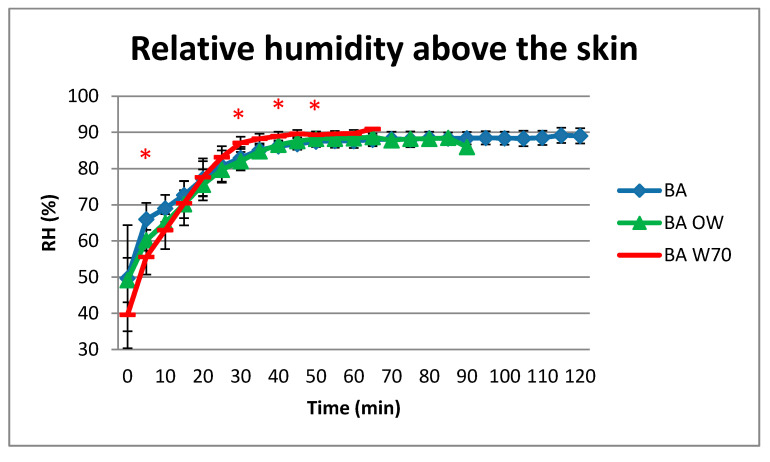
Results of average relative humidity above the skin during the physical exercise at a level of 25% VO_2max_ in air temperature of 32 °C, relative humidity of 80–85%, and air velocity of 1 m/s. * statistically significant difference, *p* < 0.05.

Results concerning the relative humidity above the skin are presented in Figure 10.

Average values of relative humidity above the skin at the end of all variants of studies were close to 90% (Figure 10). The value of 80% humidity was exceeded in 20 min for the BA and in 25 min for BA OW and BA W70. The level of moisture above the skin was statistically significantly different in the 10th min and between 30th and 50th min of the experiment for the BA and BA W70 variants, as well as between 30th and 40th min in the case of the BA OW and BA W70 variants.

### 4.6. Heat Accumulation

**Figure 11:**
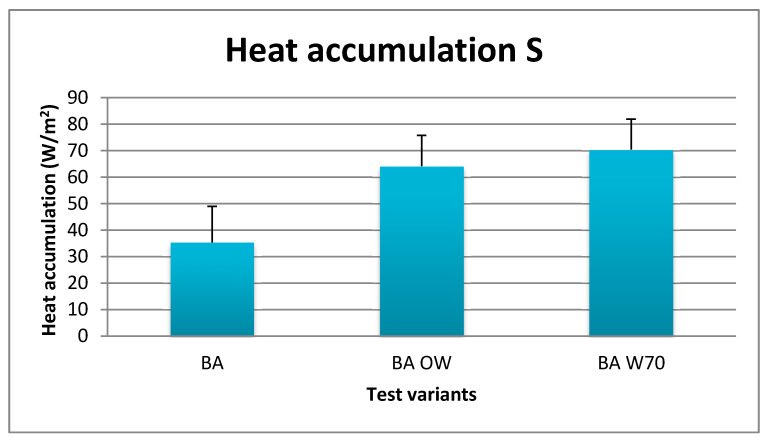
Results of average heat accumulation during physical exercise at a level of 25% VO_2max_ in air temperature of 32 °C, relative humidity of 80–85%, and air velocity of 1 m/s.

The intensity of heat accumulation is presented in Figure 11.

The lowest heat accumulation was found in tests for variant BA, at 35.4 ± 13.6 W/m^2^, the average level of accumulation was found in the protective clothing (BA OW), at 64.1 ± 11.6, but when using the W-70 apparatus the heat accumulation achieved the highest level at 70.3 ± 10.9 W/m^2^.

### 4.7. Sweating Intensity

**Figure 12:**
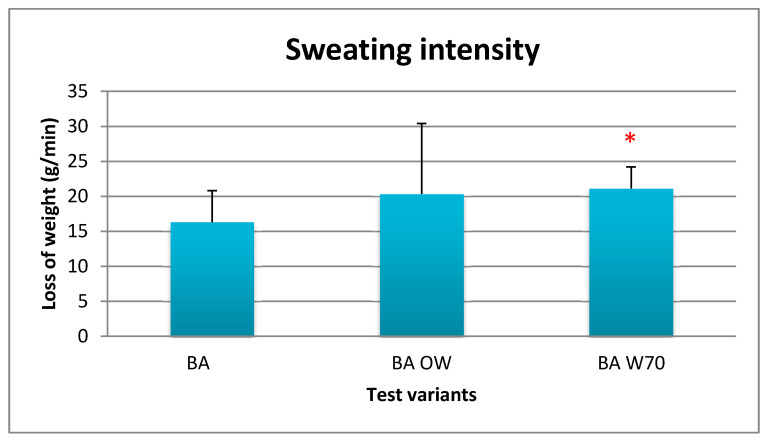
Results of average sweating intensity during physical exercise at a level of 25% VO_2max_ in air temperature of 32 °C, relative humidity of 80–85%, and air velocity of 1 m/s. * statistically significant difference, *p* < 0.05.

Due to the differences in the duration of experiment, the sweating intensity per unit of time was determined in order to unify the test results. The results of calculations are presented in Figure 12.

The lowest sweating intensity was obtained in the case of the BA variant (about 16 g/min), and the highest in the case of BA W70 (about 21 g/min). This difference was statistically significant. However, sweating intensity in the case of the BA OW variant (about 20 g/min) was similar to the result obtained in the case of the variant with the additional use of breathing apparatus.

## 5. Discussion

Physiological tests carried out with the participation of mining rescuers using both underwear and protective clothing with thermoregulation elements with PCM, carried out in difficult microclimate conditions, reflecting the typical working conditions of rescuers, were aimed at determining and comparing the heat load of users in different variants of clothing and equipment. Protective clothing and underwear with flame-retardant and anti-electrostatic properties has been specially designed for rescuers, taking into account their requirements.

The participation of mine rescuers in research was an important matter, because they are adapted to work in difficult environmental conditions and work load even at a higher level than used in the research [21]. Research has been designed to ensure rescuers’ safety, while exercising in a hot environment. The duration of tests was an indicator of safe heat load in various test variants. The longest test time was found in tests performed in the underwear (BA). The average duration of this study reached 106 min. In this variant, time of the end of experiment (120 min) was the reason for its termination three times and exceeding of the accepted internal temperature during the other three times. This fact indicates that despite the high humidity of the ambient air, heat exchange with the environment by convection and radiation was possible. It is important to note that rescuers under very harsh environmental conditions work only in the underwear to reduce the heat load associated with wearing outerwear, so for this reason the BA variant is present in tests. The properties of the developed underwear were not an obstacle to heat exchange. In such conditions the sweat intensity was not very high, as the heat accumulation was low. The level of sweating intensity per unit of time and the level of heat accumulation were the lowest in this test variant (BA) compared to the other variants. Sweating intensity per 1 h was much lower than the maximum safe level of sweating (1300 g/h according to EN ISO 9886 [20]) in the BA variant and was on the level of 960 g/h. In the other variants it was close to maximum value, reaching 1200 g/h and 1260 g/h for the BA OW and BA W70 variant, respectively.

In addition, the average internal temperature level did not reach the assumed 38.5 °C for 120 min of testing. The T_sk_ level was also the lowest in the variant BA, and it did not change from 10 min to the study’s completion. This fact indicated that the heat exchange with the environment was possible in the BA study variant. Also, the changes in HR were the mildest in variant BA; after the longest-lasting testing, the final HR value was the lowest, reaching the level of 146 beats/min. The HR level was slightly higher for the BA OW variant (151 beats/min) and similarly for the BA W70 variant, but the time of experiment was shorter: 90 min for BA OW and 65 min for BA W70. These results indicate that in the BA variant, the load on the circulatory system in thermoregulatory processes was lower than for the other two variants [8,22,23,24].

The permissible working time in the developed clothing at 32 °C was reduced to 68 min (BA OW) in relation to 106 min in the underwear (BA). Work in the protective clothing and respiratory protective equipment caused a further increase in the thermal load of rescuers; the permissible working time was reduced to 54 min. In the most difficult conditions in the presented studies (variant BA W70), rescuers made a greater effort, which was associated with wearing an oxygen apparatus, while placing these apparatus on their backs reduced the surface of the skin available for heat exchange with the environment. In this situation, the internal temperature and skin temperature increased faster, which translated into greater heat accumulation than in the BA study [23,24]. At 30 °C of air temperature, sweat is already produced to remove heat by evaporation. In difficult conditions for the evaporation of sweat, as was 85% relative humidity of the ambient air and the use of protective clothing and a breathing apparatus in the above-presented study, sweat is produced but is inefficient in the heat dissipation process, and also water loss from the body takes place [8,25].

In all tested variants, PCM elements were used in order to limit mine rescuers overheating. The weight of the introduced pouches was selected on the basis of consultations with mine rescuers and established on such level that should ensure the highest possible thermoregulation effect and at the same time be acceptable by the end users. Slight cooling mainly on the neck, chest, and back during the tests were experienced by 50% of participants, and 33% of test participants felt more intense cooling. This result confirms the positive effect of applied PCM pouches into the designed set of clothing.

Those results indicate that the developed protective clothing—despite being characterized by the minimum achievable mass, materials with very good comfort-related properties, and cooling elements—increases the rescuers heat load, as indicated by physiological parameters: internal temperature, skin temperature, heart rate, and sweating intensity. Therefore, in difficult environmental conditions in which mine rescuers work, it is extremely important to monitor the physiological responses of rescuers and keep safe working time. Moreover, in the case when the environmental conditions allow for reduction of the clothing layers, variant BA should be used.

## 6. Conclusions

The conditions of conducting rescue operations at the high air temperature and high humidity level cause a significant thermal load on the mining rescuer’s body. Analysis of the tests results of physiological parameters of rescuers performing effort at 32 °C and humidity 80–85% indicate that the equipment used by the rescuers has a large impact on this load. The use of outer clothing already causes a significant increase in the heat load of rescuers because it covers most of the skin’s surface. The addition of a respiratory protective device to this set will increase this response to a lesser extent. Totally, the observed increase of heat accumulation was at the level of 35 W/m^2^. As a consequence, the use of outer clothing shortened safe time of exposure to such conditions by about 36%, while the addition of respiratory protective device to this set shortened this time by another 13%. Hence, the slight differences in the response of rescuers between the BA OW and BA W70 study variants were observed, while the differences between BA and BA OW or BA W70 were often statistically significant. In the case of the heart rate, the differences between the tested variants reached even 30 beats/min, for the internal temperature it was equal to 0.5 °C, and for skin temperature it was more than 1.5 °C. The results of conducted tests showed that the personal protective equipment should be very carefully configured depending on the risks during rescue operations, and the number of protective equipment used should be limited if possible in order to ensure safety. Moreover, the design should allow heat and sweat to be removed from the body due to properly arranged ventilation holes and cooling elements. However, due to the threat to life of rescuers resulting from climatic conditions and physical load during rescue operations, it is necessary to monitor the working time and heat load level of rescuers to ensure their safety.

## Figures and Tables

**Table 1:** Specification of selected phase change materials (PCM) macrocapsules (production: Microtek Laboratories).

Symbol	Melting Temperature, °C	Enthalpy, J/g	Diameter, mm	Composition
MacroPCM 32	32 ± 2	160–190	3–5	80% PCM 20% polymer coating
MacroPCM 37	37 ± 2			

**Table 2:** Test variants.

Symbol	Description of a Variation of a Test
BA	Underwear with PCM elements
BA OW	Underwear with PCM elements and outer protective clothing
BA W70	Underwear with PCM elements, outer protective clothing and closed-circuit compressed oxygen breathing apparatus type W-70

**Table 3:** Number of cases for particular reasons for termination of the experiment.

Test Variant	Reason for Termination of the Experiment			
	T_abd_	HR	Subjective Reason	Time of Experiment
BA	3	-	-	3
BA OW	3	3	-	-
BA W70	2	3	1	-
